# Combination of Pectin and Eudargit RS and Eudragit RL in the Matrix of Pellets Prepared by Extrusion-Spheronization for Possible Colonic Delivery of 5-Amino Salicylic Acid

**Published:** 2013-05-04

**Authors:** Abbas Akhgari, Mohammadreza Abbaspour, Meysam Moradkhanizadeh

**Affiliations:** 1 Nanotechnology Research Center and School of Pharmacy, Ahvaz Jundishapur University of Medical Sciences, Ahvaz, IR Iran

**Keywords:** Pectin, Methylmethacrylate-methacrylic Acid Copolymer, Colonic Diseases, Drug Delivery System

## Abstract

**Background:**

Different methods have been studied for targeting drugs to the colon, such as pH-based, time dependent and bacterially degradable systems. However, due to variations in physiological conditions of patients, one system alone could not be completely reliable on colonic drug delivery.

**Objectives:**

The aim of this study was preparation and evaluation of a novel colon-specific drug delivery system for 5-ASA (mesalazine) pellets using pectin as a microbially degradable polymeric carrier and Eudragit RS (ERS) and Eudragit RL (ERL) as time-dependent polymers.

**Materials and Methods:**

Formulations were constructed based on a multilevel full factorial design. Pellets were prepared via extrusion - spheronization and evaluated for physicochemical properties, image analysis, SEM, FT-IR, DSC and in vitro drug release studies in the simulated gastric fluid with pH = 1.2 (SGF), simulated intestinal fluid with pH = 6.8 (SIF) and simulated colonic fluid with pH = 6.8 in presence of pectinolytic enzyme (SCF).

**Results:**

It was shown that in the presence of pectin, formulations without ERL had a relative resistance to drug release in SGF. Pellets containing pectin and the least amount of ERS had the highest burst release effect in SCF. On the other hand, increasing in amount of ERS in the formulations caused a sustained drug release. Presence of pectin in formulations containing ERS and ERL caused sensitivity of formulations to pectinolytic enzyme which can suitable for a colon specific drug delivery system.

**Conclusions:**

It was shown that combination of pectin and eudragits can relatively control drug release in the upper GI. On the other hand, pectin degraded in the presence of pectinase and formulations were susceptible to the colonic media.

## 1. Background

Extrusion / spheronization is one of the most important methods in pellet production. Pellets that produce by this method have benefits like narrow range of particle size, regular shape (1), maximizing drug absorption, and reducing risk of dose dumping (2). There are some variables in this technique like mixing method and time, type and amount of granulation liquid, type of extruder and spheronizer, time of spheronization, etc. Selection of each variable has a great effect on the yielded pellets (3). Pelletization of hydrophilic polysaccharides by extrusion-spheronization has not been very well established. Chatchawalsaisin *et al.* used a combination of two types of hydrophilic polymers, sodium alginate and chitosan in the formulation of pellets prepared by extrusion - spheronization to assess their ability to produce suitable pellets. They reported negligible benefit in using a combination of the two polymers in comparison of sodium alginate alone for sustaining drug release (4). In another study three types of pectin including high methoxylated, low methoxylated, and amidated low methoxylated pectin and different concentrations of granulation liquids (*i.e.* methanol, ethanol, citric acid, lactic acid, and calcium chloride) were used to produce pellets. It was reported that type and concentration of added granulation liquid and type of pectin affect the characteristics of pellets such as sphericity and moisture content (5). Degree of amidation of pectin and concentration of calcium ions had also a considerable effect on pellet sphericity (6). Colon - specific drug delivery systems can deliver drugs to the colon for the treatment of colonic diseases such as IBD, colorectal cancers and Crohn's disease (7, 8). There are different approaches for targeting drugs to the colon. The main systems are pH-based, time dependent, and bacterially degradable. However, regarding the variation in physiological conditions of people, one system alone could not be completely reliable on colonic drug delivery. Therefore, works have been notably performed with a combination of the aforementioned systems. Some instances are combination of pH- and time-dependent systems for a pulsatile capsule (9), poly(l-lactide-co-glycolide) and Eudragit S-100 (10), Eudragit RS and Eudragit L 100 / Eudragit S 100 (11), time-dependent and enzyme-degradable polymers (12, 13), and enzyme-degradable polymers and pH - dependent polymetacrylates (13). However, preparation of multiparticulates such as pellets for colon-specific drug delivery using a system based on time-dependent polymetacrylates and enzyme degradable polysaccharides has not been established.

## 2. Objectives

Therefore, the objective of this study was to produce and evaluate a novel colon specific drug delivery system for 5-ASA matrix pellets using a mixture of Eudragit RS and Eudragit RL as time - dependent polymers and pectin as a bacterially degradable polysaccharide.

## 3. Materials and Methods

### 3.1. Materials

High methoxylated pectin (Obipectin ®, Methoxylation 70%, Bishofszell, Switzerland), 5-ASA (Arya, Iran), microcrystaline cellulose (BioPolymer, Ireland), Eudragit RS PO and Eudragit RL (GmbH, Germany), and pectinolytic enzyme (pectinase from aspergillus niger, Sigma, Germany) were purchased from indicated sources.

### 3.2. Factorial Design

Formulations were produced based on a multilevel full factorial design. The studied independent variables were a ratio of Eudragit RS: Eudragit RL (X_1_), an amount of pectin (X_2_) and an amount of Eudragits (X_3_) in formulations. Types and levels of the independent variables are listed in (Table 1). Dependent variables (responses) were: mean dissolution time (MDT) of pellets in HCl with pH = 1.2 (Y_1_), in media with pH = 6.8 without enzyme (Y_2_) and in media with pH = 6.8 with enzyme (Y_3_), and drug release at 30 minutes in the media with pH = 6.8 without (Y_4_) and with enzyme (Y_5_). Details of all the formulations (runs) based on experimental design are illustrated in (Table 2). Formulations F_9_ and F_18_ were not practically spheronizable and thus were removed from list of runs.

**Table 1.** Independent Variables: Types and Levels

Variable	Levels		
**X_1_: Ratio of EudragitRS : Eudragit RL**	1:0	1:1	-
**X** **_2_** **: Percent of pectin in the formulation**	0%	10%	20%
**X** **_3_** **: Percent of Eudragit in the formulation**	10%	20%	30%

**Table 2.** Composition of Experimental Formulations (all Formulations Contain 20% Drugs)

Run	Eudragit RS, %	Eudragit RL, %	Pectin, %	Microcrystalline Cellulose, %
**1**	10	0	0	70
**2**	20	0	0	60
**3**	30	0	0	50
**4**	10	0	10	60
**5**	20	0	10	50
**6**	30	0	10	40
**7**	10	0	20	50
**8**	20	0	20	40
**9**	30	0	20	30
**10**	5	5	0	70
**11**	10	10	0	60
**12**	15	15	0	50
**13**	5	5	10	60
**14**	10	10	10	50
**15**	15	15	10	40
**16**	5	5	20	50
**17**	10	10	20	40
**18**	15	15	20	30

### 3.3. Preparation of Pellets

5-ASA (20%) and different percent of pectin, Eudragit RS PO, Eudragit RL PO and microcrystalline cellulose (MCC) were mixed using a kitchen mixer for 10 minutes and sufficient amount of granulating liquid (ethanol/water 70:30) was added to the mixture. Pellets were prepared via extrusion - spheronization using a screw extruder (type HC 732, Dorsa, Iran) with a 1 mm screen at 100 rpm. The extrudates were spheronized using a spheronizer (type HC 732, Dorsa, Iran) with crosshatched plate 1000 rpm for 10 minutes. The pellets that were dried in an oven (typeWV 30 UL, memmert, GmbH, Germany) at 50°C for 3 h.

### 3.4. Characterization of Pellets

#### 3.4.1. Sieve Analysis

Formulations were sieved using a set of sieves with different mesh sizes (14, 20, 35, 50 and 60) and shaken with a sieve shaker (type AR400, Erweka, GmbH, Germany) for 5 minutes. The pellets that remained on the sieve with mesh 35 were considered as proper pellets.

#### 3.4.2. Image Analysis

Shape and size of pellets were examined by microscopic image analysis. About 20 pellets from each batch were attached on a black background. Image analyzer consisted of a stereomicroscope (Olympus, DP25, Okura, Japan) at magnification (× 80) and a camera (Olympus, E620, Okura, Japan). Obtained images were analyzed by sicon image analyzing software (Scion Image for Windows, Release Beta 4.0.2). The area (A), perimeter (p_m_) and Feret diameters of pellets (d_max_ and d_min_) were measured and two shape factors were calculated as followed:

(1) *Aspect ratio* = d_max_ / d_min_

(2) *Sphericity* = 4πA / P_m_^2^

#### 3.4.3. Scanning Electron Microscopy

Surface characteristics of pellets were studied on some formulations as samples using a scanning electron microscope (KYKY, China). Samples were selected from intact pellets, pellets after immersion for 5 h in phosphate buffer solution with pH = 6.8 without pectinolytic enzyme and pellets after immersion for 5 hours in phosphate buffer solution with pH = 6.8 in presence of pectinolytic enzyme.

#### 3.4.4. FT-IR

FTIR spectroscopy was accomplished in KBr discs over a range of 4000–500 cm-1 using IR spectroscope equipment (vortex 70, Bruker, Germany). FTIR studies were performed to assess possible interaction between drug and polymers.

#### 3.4.5. Differential Scanning Calorimetry

To assay purity and physical properties of samples and survey interaction between component of formulations, DSC studies were carried out on some formulations as samples using DSC equipment (Mettler, Toledo, Switzerland) at the rate of 10°C/minutes at the temperature range between 25 to 350°C.

### 3.5. Dissolution studies

Dissolution studies were performed with 200 mg of pellets in a dissolution tester (DT800, Erweka, Germany) using USP apparatus I, at a rate of 100 rpm and 37◦C, in 900 mL of dissolution medium. For simulating various parts of GI tract, different media were used including HCl solution with pH = 1.2 simulating gastric fluid (SGF), phosphate buffer with pH = 6.8 with simulating intestinal fluid (SIF) and phosphate buffer with pH = 6.8 containing pectinolytic enzyme with concentration of 0.6 mg/mL simulating colonic fluid (SCF). Samples were taken and the released drug from pellets was assayed sphectophotometrically by a UV / Visible spectrophotometer (Biowave II, WPA, England) at a wavelength of 302 and 320 nm for acidic and buffer media, respectively. From drug release data, mean dissolution time (MDT) was computed via following equations (14):

**(3)***MDT* = ∑t_i_^-^ × ∆M_i_ / ∑∆M_i_

**(4)** t_i_^-^ = t_i_ + t_i + 1 _/ 2

**(5)** ΔM_i_ = M_i + 1 - M_i__

ΔM_i_ is the amount of drug had been released from formulation in the time period between t_i_ and. t_i + 1_

### 3.6. Statistical Analysis of Data

Impact of independent variables (X) on the dependent variables (Y) revealed using a polynomial second order equation as followed:

**(6)** Y = C + b_1_X_1_ + b_2_X_2_ + b_3_X_3_ + b_4_X_1_^2^ + b_5_X_2_^2^ + b_6_X_3_^2^ + b_7_X_1_X_2_ + b_8_X_1_X_3_ + b_9_X_2_X_3_

The models were simplified with a backward, stepwise linear regression technique by SPSS version 14 and significant expressions (*P* < 0.05) were selected for the final model Surface plots related to each equation depicted by Statgraphics version Centurion XVI.

## 4. Results

**Figure 1.**
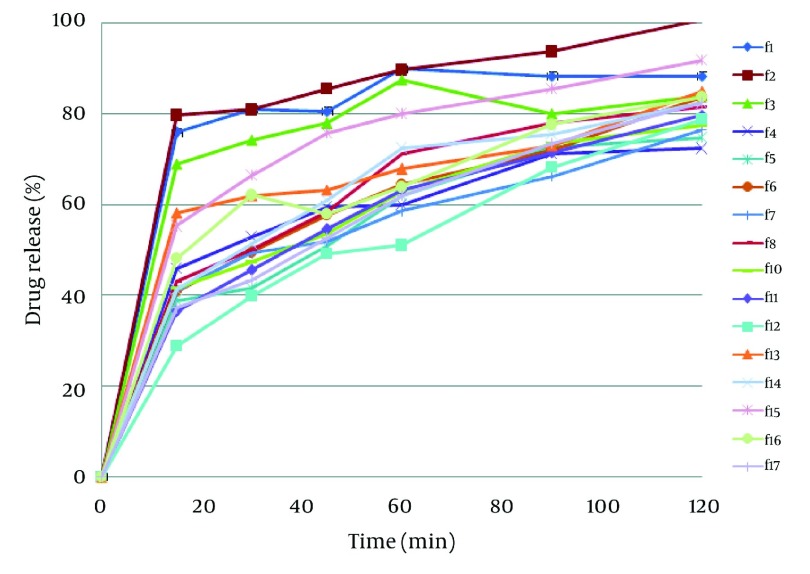
Dissolution Profile of Formulations in the Acidic Media

**Figure 2.**
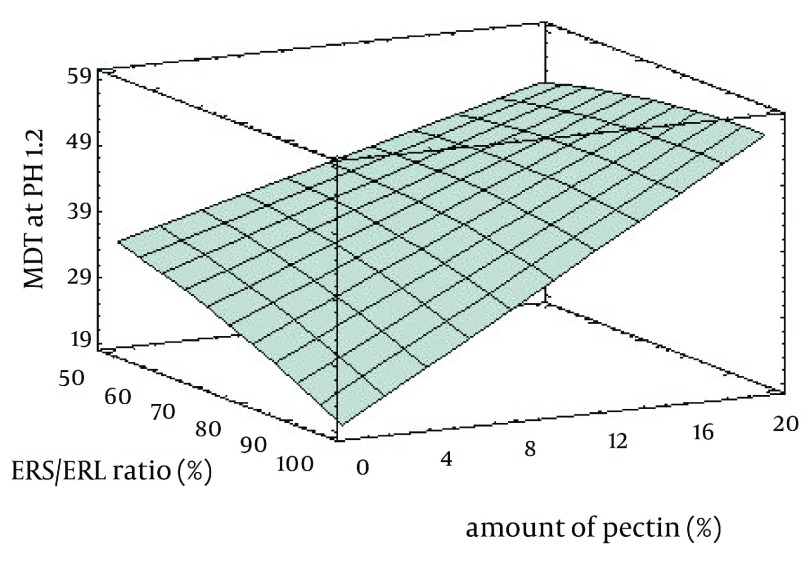
Influence of Amount of Pectin and ERS/ERL Ratio on the MDT of Pellets at pH = 1.2

Results of image analysis are depicted in Table 3. Sphericity and aspect ratio of pellets are near to 1 and therefore spherical pellets had been obtained by extrusion - spheronization. Data of drug release in the media with pH = 1.2 (Figure 1) showed a burst drug release from all formulations after 15 minutes and the most burst drug release belonged to formulations F_1_, F_2_ and F_3_ (*P* < 0.05). These formulations did not have ERL and pectin in their compositions and compared to the other formulations their drug release reached a plateau following initial burst release. Eudragit RS and RL have quaternary groups in their structure responsible for hydration and swelling of polymer. These functional groups are conjugated with the chloride anion. Therefore, in the presence of the extra chloride ion, possibility of ion exchange needed for hydration would be more restrictive (15). Regarding more amounts of ammonium groups in ERL compared with ERS, swelling in the acidic media could be lowered by addition of ERL in the formulations. On the other hand, controlling drug release in media with pH = 1.2 by pectin is compatible with the findings of other studies and it could be explained that pectin contains carboxylic functional groups which are not ionized in the acid and can potentiate resistance to drug release (16, 17). Figure 2 shows the response surface plot of response Y1 (MDT of pellets at pH = 1.2). The controlling effect of pectin on drug release in the acid medium is obvious in Figure 2.

**Table 3.** Sphericity and Aspect Ratio of Pellets

Run	Aspect ratio	Sphericity
**1**	1.263322	0.746609
**2**	1.280731	0.699653
**3**	1.317182	0.667813
**4**	1.238995	0.715607
**5**	1.207559	0.776522
**6**	1.235942	0.771249
**7**	1.172391	0.738544
**8**	1.317183	0.57675
**10**	1.258226	0.753068
**11**	1.199188	0.8247
**12**	1.370763	0.77773
**13**	1.584275	0.653902
**14**	1.369667	0.634178
**15**	1.35841	0.690521
**16**	1.210077	0.561392
**17**	1.173363	0.660059
**18**	1.28483	0.703019

**Figure 3.**
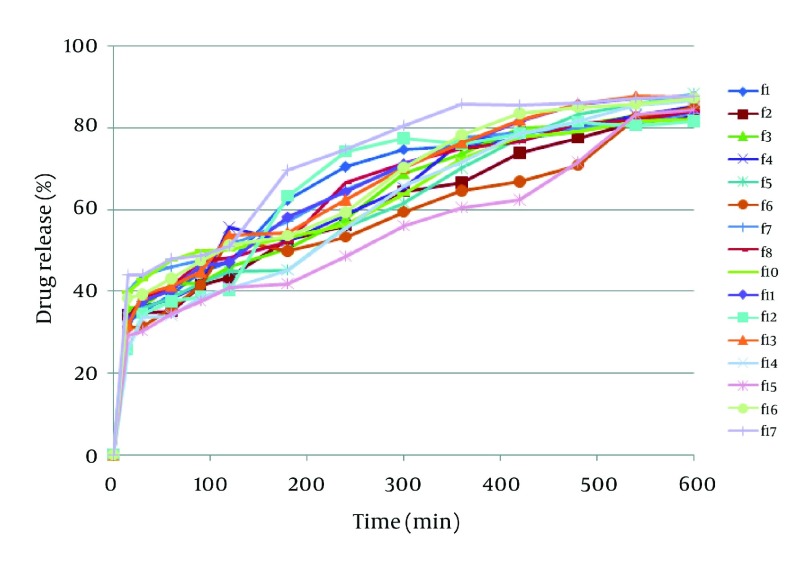
Dissolution Profile of Formulations at pH = 6.8 Without Enzyme

**Figure 4.**
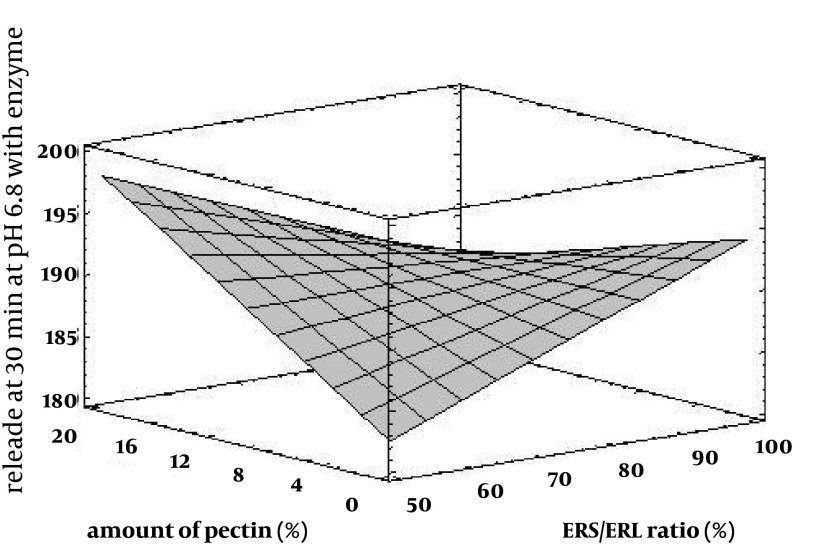
Influence of Amount of Pectin and ERS/ERL Ratio on the Drug Release at 30 Minutes in pH = 6.8 With Enzyme

**Figure 5.**
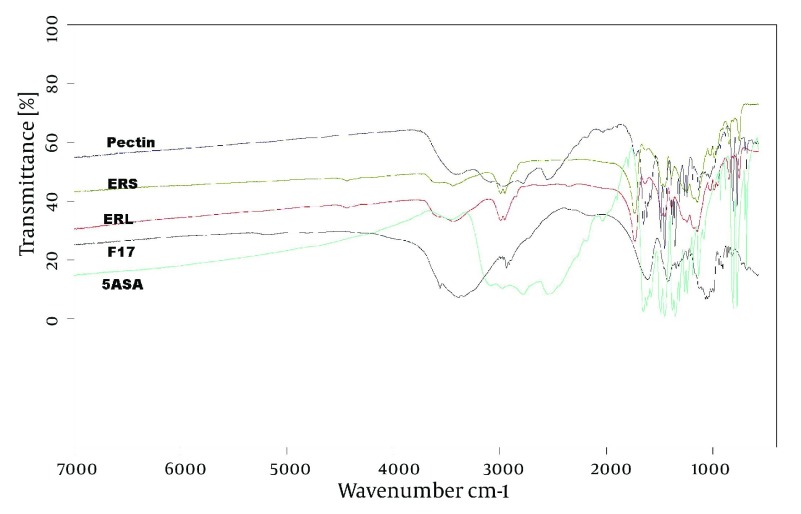
FTIR Diagrams of Materials Used in Formulations

**Figure 6.**
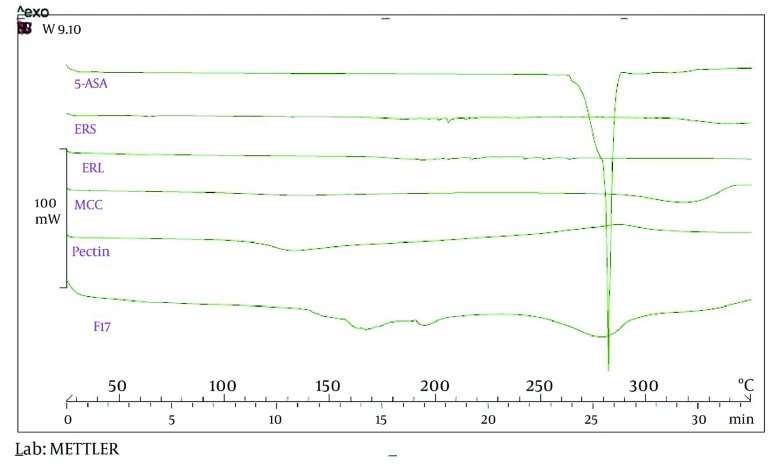
DSC Thermograms of Materials Used in Formulations

Drug release profile in the buffer media with pH = 6.8 in the absence of pectinolytic enzyme (Figure 3) revealed a burst drug release for all formulations at 15 minutes after which a linear sustained drug release occurred which could be due to the action of time-dependent swelling of ERS and ERL in the matrix of pellets. As shown in Figure 4, the acidic media drug release at pH = 6.8 was increased by addition of ERL and pectin in the composition of pellets which could be due to greater permeability of aforementioned polymers in comparison with ERS and lack of ion exchange restriction in the buffer media. Also, according to FTIR diagrams (Figure 5) there was no possible interaction between these polymers which could affect drug release. DSC diagrams (Figure 6) also confirmed the lack of significant interaction between polymers (and drug), melting peak of drug could be seen in the same region as pure drug but alteration in the shape of melting peak in pellet formulation could be attributed to lower amount and decreasing the crystallinity of drug in pellet formulations. The slight shift of a peak from 130 to 170°C in F_17_ compared with pectin powder could be due to the difference of bound water between pectin in powder and mucilage form.

**Figure 7.**
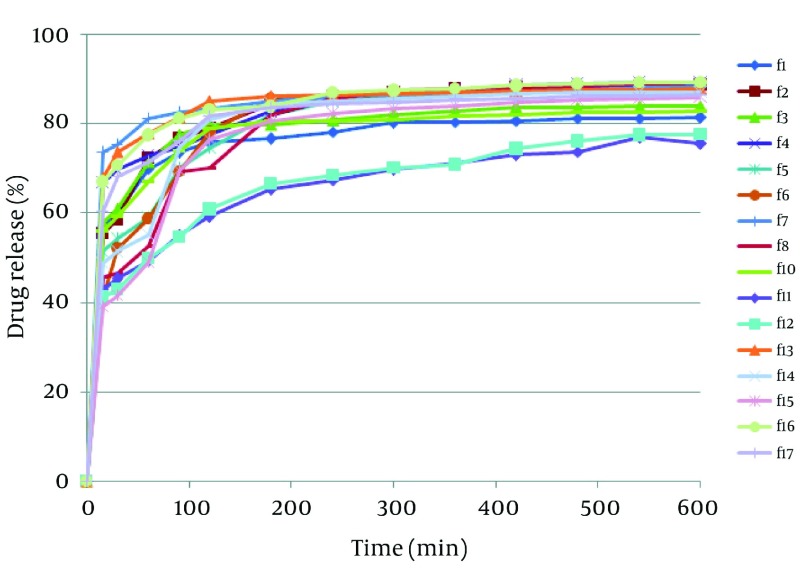
Dissolution Profile of Formulations at pH = 6.8 With Enzyme

**Figure 8.**
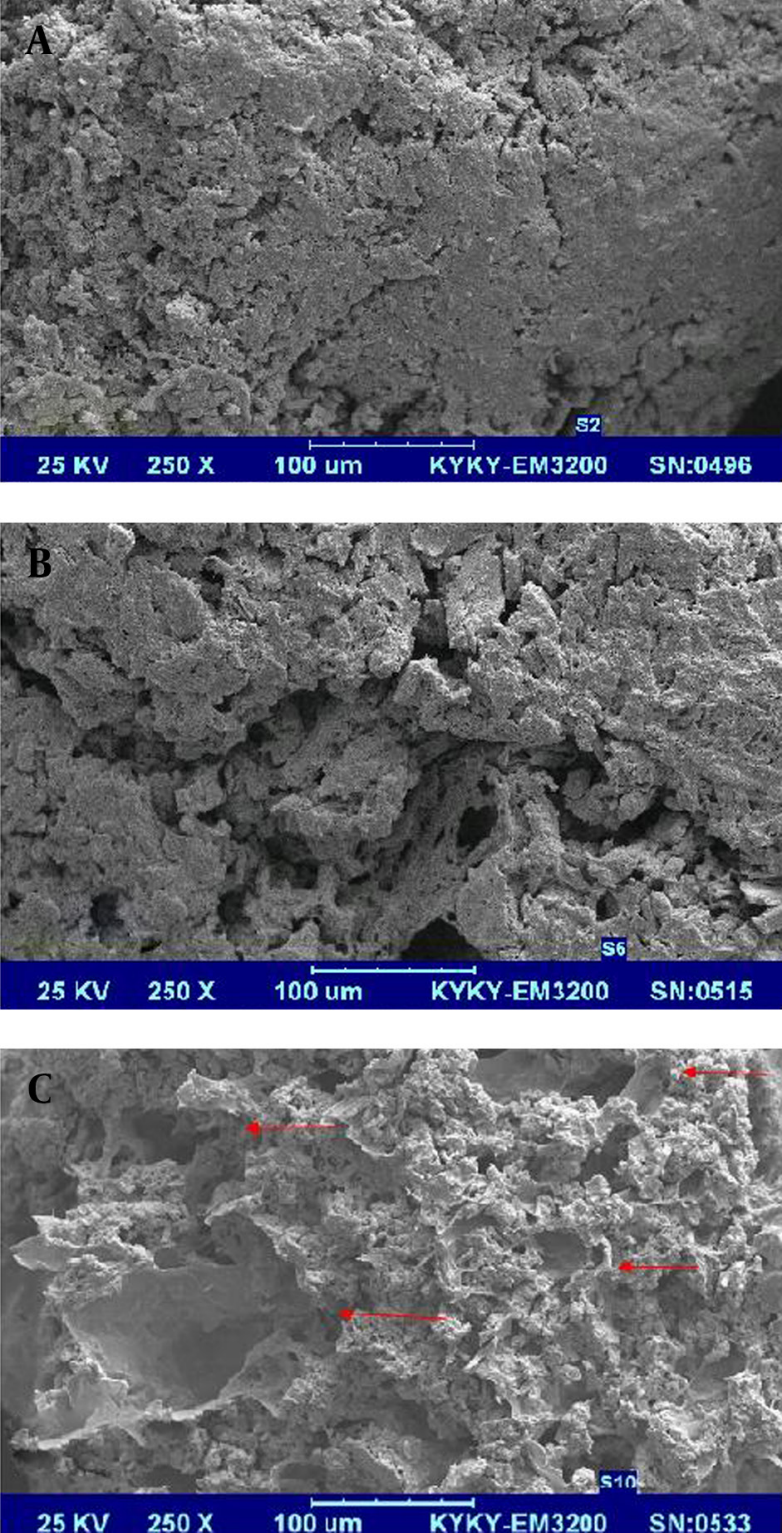
Scanning Electron Micrograph of Surface of Pellets (Formulation F_17_) **A.** Dry pellet, **B.** After 5 hours immersion in buffer with pH = 6.8, **C.** After 5 hours immersion in buffer with pH = 6.8 with enzyme (magnification 250 ×)

Figure 7 illustrates drug release from pellets in the buffer media with pH = 6.8 in the presence of pectinolytic enzyme. According to this figure formulations F_11_ and F_12_ which contained ERS and ERL and did not have pectin in their composition showed slowest burst drug release followed by sustained release which is due to the presence of time-dependent polymethacrylates in pellet compartments (18) , whilst formulations F_4_, F_7_, F_13_, F_16_ and F_17_ released the drug immediately. The latter formulations contained 10-20% of pectin. Pectin is a natural polysaccharide isolated from sources like citrus and apple and depending on degrees of methoxylation can be categorized into high (HM) and low methoxy (LM) pectin (18). The type of pectin used in this investigation was HM which is less water soluble compared to pectin LM (19). However, it has not enough resistance to dissolution in the upper GI tracts and its combination with sustained release polymers ERS and ERL would be necessary to prevent premature drug release. On the other hand, pectin is susceptible to pectinolytic enzyme presented in the colonic fluid which could have an effect on the basic backbone of this polysaccharide and manage its degradation (20). Ugurlu and coworkers suggested that in the combination of pectin with a swellable polymer the hydration of polymers could be critical for accessibility of pectin for degradation (21). In our study, hydration and further swelling of ERS and/or ERL could achieve this result and breakdown of pectin backbones due to the effect of pectinolytic enzyme and pore creation in matrices of pellets illustrates more drug release from formulations containing pectin. SEM images (Figure 8) confirmed degradation of pectin in simulated colonic fluid and the pores resulted from pectin leaching are obvious in Figure 8.

Therefore, formulations containing pectin can be susceptible to colonic media and drug release from pellets in the colon would be more likely to occur. Using the other method, the presence of Eudragit RS and Eudragit RL relatively controlled drug release in the upper GI tracts. To prevent more premature drug release from formulations in the stomach, enteric coating of pellets may be useful.

## 5. Discussion

Pellets of 5-ASA using pectin and Eudragit RS and Eudragit RL prepared and their properties such as sphericity, aspect ratio and drug release profile were investigated. It was demonstrated that in the simulated gastric media pectin can hinder drug release. Drug release was also controlled by eudragits in buffer media but in the simulated colonic fluid, pectin degraded to its monomers due to degradation by pectinolytic enzyme followed by pore creation and relatively fast drug release. Therefore, pellets with the suitable characteristics for colonic delivery of 5-ASA could be achieved by extrusion-spheronization of optimized mixture of pectin and eudragit RS and eudragit RL. Further in vivo studies of manufactured formulations are needed to produce a practical novel colon specific drug delivery system.
